# Polyethylene glycol-modified dendrimer-entrapped gold nanoparticles enhance CT imaging of blood pool in atherosclerotic mice

**DOI:** 10.1186/1556-276X-9-529

**Published:** 2014-09-26

**Authors:** Kaichuang Ye, Jinbao Qin, Zhiyou Peng, Xinrui Yang, Lijia Huang, Fukang Yuan, Chen Peng, Mier Jiang, Xinwu Lu

**Affiliations:** 1 Department of Vascular Surgery, Shanghai Ninth People's Hospital Affiliated to Shanghai Jiao Tong University, School of Medicine, Shanghai 200011, People's Republic of China; 2 Department of Radiology, Shanghai Tenth People's Hospital Affiliated to Tongji University, School of Medicine, Shanghai 200072, People's Republic of China; 3 Vascular Center of Shanghai Jiao Tong University, Shanghai 200011, People's Republic of China

**Keywords:** Atherosclerosis, Macrophages, Gold nanoparticles, Dendrimers, Computed tomography imaging

## Abstract

We report a new use of dendrimer-entrapped gold nanoparticles (Au DENPs) modified by polyethylene glycol (PEG) with good biocompatibility for *in vitro* and *in vivo* imaging of atherosclerotic mice by computed tomography (CT). In this study, Au DENPs were synthesized using poly(amidoamine) (PAMAM) dendrimers of generation 5 (G5.NH_2_) modified by PEG monomethyl ether (G5.NH_2_-*m*PEG_20_) as templates. *In vitro* cytotoxicity and flow cytometry assays show that the formed PEGylated Au DENPs have good biocompatibility and are non-cytotoxic at the Au concentration up to 300 μM. Silver staining and transmission electron microscopy (TEM) further confirm that the Au DENPs are able to be uptaken by macrophages and are located dominantly in the lysosomes of the cells. Importantly, the formed PEGylated Au DENPs are able to be used for CT imaging of murine macrophages *in vitro* and macrophages in atherosclerotic mice *in vivo* using apolipoprotein-E-gene-deficient mice as a model. These findings suggest that the formed PEGylated Au DENPs are a promising contrast agent for CT imaging of atherosclerosis.

## Background

Atherosclerosis and its complications are the leading causes of morbidity and mortality in modern societies [1,2]. Atherosclerosis is a processed disease, characterized by endothelial injury, lipid deposition, inflammation, and plaque formation [3-6]. Among the complications, ruptures of vulnerable atherosclerotic plaques are the major cause of death in cardiovascular diseases [7,8]. Thus, the enhancement of early detection and identification of patients who are prone to plaque rupture is highly important. Recent studies have emerged to show the importance of macrophages in the formation and progression of atherosclerosis [9,10], and the high macrophage content is one of the principal factors associated with plaque instability [11]. Thus, new platforms and technologies are crucial for noninvasive imaging and assessment of the macrophage burden in atherosclerotic plaques.

The emergence of molecular imaging (MI) has drastically changed medical science in many ways, including the diagnosis of diseases in the early stages, the assessment of risks, the treatment outcome, and the follow-up [12-14]. Computed tomography (CT), one of the most reliable MI modalities, is widely used in cardiovascular diseases because of its properties of high resolution, sharp contrast, three-dimensional (3D) capabilities, and low cost in comparison to other imaging modalities [15-17]. Numerous studies showed that the key element in CT imaging of cardiovascular diseases is to design applicable and safe contrast agents with long imaging time, sharp contrast sensitivity, and excellent stability *in vitro* and *in vivo*[18,19]. Given that clinically used ionic or non-ionic CT contrast agents have several drawbacks such as allergic reaction, short imaging time, renal toxicity at high concentrations, and nonspecificity [20,21], developing novel nanoparticle (NP)-based contrast agents with excellent biocompatibility and prolonged blood circulation time is crucial for CT imaging.

Gold nanoparticles (AuNPs), one of the most promising nanomaterials, are widely used as X-ray CT imaging contrast agents in the diagnosis and therapy of tumors because of their *in vitro* and *in vivo* stability, easy surface modification, good biocompatibility, and higher X-ray attenuation characteristics than those of clinically used ionic or non-ionic iodinated CT contrast agents [17,22]. However, to develop AuNP-based CT contrast agents with desirable properties, such as good biocompatibility, easy and controllable surface modification, sharp contrast, and prolonged imaging time [23-25], multiple complicated steps are generally required. Recent studies have shown that proper modification of AuNPs can extend their circulation time in cardiovascular diseases by decreasing the rapid uptake and clearance by the reticuloendothelial system (RES) [19,26-28]. One promising and efficient technique to achieve this goal is to modify AuNPs with polyethylene glycol (PEG) molecules [29,30].

In our previous reports [31,32], we have shown that acetylated dendrimer-entrapped AuNPs (Au DENPs) synthesized using generation 5 (G5) poly(amidoamine) (PAMAM) dendrimers as templates are able to be used as contrast agents for CT imaging of C57 mice after intravenous injection. X-ray absorption coefficient measurements verified that the X-ray attenuation of Au DENPs was significantly higher than that of clinically used iodinated contrast agents at the same concentration (Au versus iodine) [32,33]. The inferior vena cava (IVC) and pulmonary veins of the mice were clearly identified after intravenous injection of the Au DENPs. In our another work [29], we have shown that Au DENPs formed using PEGylated G5 dendrimers as templates have improved biocompatibility and are able to be used as contrast agents for blood pool and tumor CT imaging. These prior successes lead us to hypothesize that PEGylated Au DENPs can also be used to image blood pool in atherosclerotic mice *in vitro* and *in vivo*.

In this study, PEGylated Au DENPs were generated using G5 PAMAM dendrimers modified with PEG as templates. The formed PEGylated Au DENPs were systematically assessed in terms of their stability, biocompatibility, X-ray attenuation characteristics, and feasibility as contrast agents for CT imaging of murine macrophages in atherosclerosis. The biocompatibility of PEGylated Au DENPs was evaluated by cell counting kit-8 (CCK-8) assay for cell viability and flow cytometry for cell apoptosis. The distribution of Au DENPs within macrophages associated with atherosclerosis was observed by silver staining and transmission electron microscopy (TEM). Then, the PEGylated Au DENPs were used to diagnose the atherosclerotic mice model successfully. To our knowledge, this is the first report related to the PEGylated Au DENPs used as contrast agents for CT imaging of blood pool in atherosclerotic mice *in vitro* and atherosclerotic mice model *in vivo*.

## Methods

### Materials

Ethylenediamine core amine-terminated G5 dendrimers (G5.NH_2_) with a polydispersity index less than 1.08 were purchased from Dendritech (Dendritech Inc., Midland, MI, USA). PEG monomethyl ether with one end of maleimide (mPEG-MAL) was from Shanghai Yanyi Biotechnology Corporation (Shanghai, China). All other chemicals were obtained from Aldrich (Sigma-Aldrich, St. Louis, MO, USA) and used as received. Ana-1 cell line (a mouse macrophage cell line) was purchased from Shanghai Cell Bank, the Chinese Academy of Sciences (Shanghai, China). Penicillin, streptomycin, and fetal bovine serum (FBS) were purchased from Sigma (Sigma-Aldrich, St. Louis, MO, USA). Trypsin-EDTA, Dulbecco's PBS, RPMI 1640 medium, and bovine serum albumin were obtained from GIBCO-BRL (Life Technologies, Gaithersburg, MD, USA). The water used in all the experiments was purified using a Milli-Q Plus 185 water purification system (Millipore, Bedford, MA, USA) with a resistivity higher than 18 mΩ cm. Regenerated cellulose dialysis membranes (molecular weight cutoff, MWCO = 10,000) were acquired from Fisher (Thermo Fisher Scientific, Waltham, MA, USA).

### Synthesis of [(Au^0^)_300_-G5.NHAc-*m*PEG] DENPs

The procedure used to synthesize [(Au^0^)_300_-G5.NHAc-*m*PEG] DENPs was adopted from our previous report [29]. The solution of G5.NH_2_-*m*PEG and HAuCl_4_ (molar ratio between G5.NH_2_-*m*PEG and HAuCl_4_ is 1 to 300) was mixed for 30 min, followed by an addition of NaBH_4_ (four times molar excess of the Au salt) solution (V(H_2_O):V(CH_3_OH) = 1:1) under vigorous stirring for 2 h to obtain a wine red solution (denoted as [(Au^0^)_300_-G5.NH_2_-*m*PEG] DENPs).

Then, triethylamine (five times molar excess of the total primary amines of G5.NH_2_) was added to the aqueous solution of [(Au^0^)_300_-G5.NH_2_-*m*PEG] DENPs while under magnetic stirring. After 30 min, acetic anhydride (four times molar excess of the total primary amines of G5.NH_2_) was added to the above mixture solution under stirring, and the mixture was allowed to react for 24 h. The final reaction mixture solution was extensively dialyzed against PBS buffer (three times, 4 L) and water (three times, 4 L) for 3 days to remove the excess reactants and by-products, followed by lyophilization to obtain the PEGylated Au DENPs ([(Au^0^)_300_-G5.NHAc-*m*PEG] DENPs).

### Characterization techniques

UV-vis spectra were obtained using a Lambda 25 UV/Vis spectrophotometer (Perkin Elmer, Waltham, Massachusetts, USA). Samples were dissolved in water before the experiments. TEM was performed using a JEOL 2010 F analytical electron microscope (JEOL Ltd., Akishima-shi, Japan) operating at 200 kV. An aqueous solution of [(Au^0^)_300_-G5.NHAc-*m*PEG] DENPs (5 μL, 1 mg/mL) was dropped onto a carbon-coated copper grid and air-dried prior to measurements.

### Cell culture and characterization

Murine macrophages Ana-1 cells were cultured in RPMI 1640 medium with 10% heat-inactivated FBS and 1% *v*/*v* penicillin/streptomycin at 37°C and 5% CO_2_. Immunofluorescent staining was performed to characterize the Ana-1 cells. Ana-1 cells were fixed with 4% paraformaldehyde for 20 min at 37°C. After permeabilization in 0.3% Triton-X100 (diluted in PBS), the cells were incubated with 10% goat serum albumin diluted in 0.5% bovine serum albumin (BSA) for 30 min at 37°C to block nonspecific antibody adhesion. The primary macrophage antibodies F4/80 (rat monoclonal, 1:500 diluted in 0.5% BSA; Abcam, Cambridge, UK) and MAC-3 (rat monoclonal, 1:500 diluted in 0.5% BSA; BD, Franklin Lakes, NJ, USA) were incubated at 4°C overnight. After rinsing with PBS, Alexa Fluor® 555 (goat anti-rat, 1:500 diluted in 0.5% BSA; Invitrogen, Grand Island, NY, USA) was applied and incubated for 1 h at 37°C in the dark. After further washing with PBS, the cell nuclei were counterstained with 4′,6-diamidino-2-phenylindole (DAPI) (1:500, diluted in 0.5% BSA, DAKO, Real Carpinteria, CA, USA) for 20 s. After a final washing with PBS, the cells were examined under fluorescence microscope (Nikon, Chiyoda-ku, Tokyo, Japan).

To characterize the phenotypes of Ana-1 cells, 1 × 10^6^ cells were washed with PBS and incubated with phycoerythrin (PE)-conjugated anti-mouse antibodies against F4/80 for 30 min at 4°C in the dark. Isotype control antibodies were used as the control group (all from eBioscience, San Diego, CA, USA). After washing, the cells were analyzed by a flow cytometer (Beckman Coulter, Fullerton, CA, USA).

### Cytotoxicity assay of PEGylated Au DENPs

The viability of macrophages treated with PEGylated Au DENPs was evaluated via CCK-8 (Dojindo Laboratories, Kumamoto, Japan) assay. The assays were carried out according to the procedure described in a previous report [34]. In brief, Ana-1 cells were seeded in 96-well culture plates at a density of 1 × 10^5^ cells per well in quadruplicate and allowed to grow for 24 h. Then, the medium was replaced by RPMI 1640 medium containing PEGylated Au DENPs with different concentrations (0 to 4 μM), and the cells were incubated for 24 h at 37°C. After treatment, the medium was aspirated and cells were washed with PBS thrice. Then, 10 μL of CCK-8 diluted in 100 μL of fresh RPMI 1640 medium was added to each well and the cells were incubated for additional 2 h at 37°C. The absorbance was measured at 450 nm. The assay was carried out according to the manufacturer's instructions. For each concentration of Au DENPs, mean and standard deviation of the quadruplicate wells were reported.

The cytotoxicity of PEGylated Au DENPs was further examined by flow cytometric detection of cell apoptosis. Ana-1 cells were seeded in 6-well plates at a density of 2 × 10^6^ cells per well in quadruplicate and grown to confluence for 24 h. The medium was replaced with a fresh medium containing PEGylated Au DENPs with two different concentrations (1 and 3 μM), and the cells were incubated for 24 h. Then, the cells were harvested, washed with PBS, fixed in 0.5% paraformaldehyde citrate buffer for 2 h, centrifuged, and resuspended with PBS at a cell density of 1 × 10^6^ cells/mL. The cell suspensions were stained with Annexin V for apoptosis tests and analyzed by Cell Quest software (FACS Calibur, BD, Franklin Lakes, NJ, USA).

### *In vitro* micro-CT imaging of macrophages

Ana-1 cells were seeded in 6-well plates at a density of 2 × 10^6^ cells per well in triplicate and allowed to grow to 80% to 90% confluence for 24 h. Then, Ana-1 cells were incubated with fresh medium containing PEGylated Au DENPs (0, 100, and 300 μM, respectively) for 4 h at 37°C. After washing with PBS, cells were trypsinized, centrifuged, and resuspended with 100 μL PBS in a 0.5 mL Eppendorf tube containing approximately 4.0 × 10^6^ Ana-1 cells for each tube. The cell suspension in each tube was placed in a self-designed scanning holder and then scanned using a micro-CT imaging system (eXplore Locus, GE Healthcare, London, Ontario, Canada) with the following operating parameters: tube voltage, 80 kV; tube current, 450 μA; exposure time, 400 ms; slice thickness, 45 μm; and scan field of view, 45 × 80 mm. Images were reconstructed on a micro-CT imaging workstation (GEHC microView, GE Healthcare, London, Ontario, Canada) using the following parameters: voxel, 45 × 45 × 45 μm; display field of view, 10 × 25 mm. CT values were acquired on the same workstation using the software supplied by the manufacturer. Each experiment was carried out in triplicate.

### Cellular uptake of PEGylated Au DENPs

The cellular uptake of PEGylated Au DENPs was verified by silver staining and TEM according to methods reported in our previous work [17,29]. For silver staining experiments, Ana-1 cells were seeded at a density of 2 × 10^6^ per well in 6-well plates inserted with polylysine-coated cover slips for 24 h and treated with fresh medium containing PEGylated Au DENPs (1 and 3 μM) for 24 h at 37°C. After washing with PBS, the cells were fixed in 4% paraformaldehyde solution for 2 h. Silver staining was performed according to the manufacturer's instruction. Cells were washed with PBS thrice, stained with 1% nuclear fast red to stain the cell nuclei, washed, air-dried, dehydrated, cleaned, and mounted for light microscope observation.

For TEM imaging of the cellular uptake of PEGylated Au DENPs, Ana-1 cells were seeded at a density of 2 × 10^6^ per well in 6-well plates for 24 h to grow to 90% confluence and treated with a fresh medium containing PEGylated Au DENPs (3 μM) for 24 h at 37°C. After washing thrice with PBS, the cells were trypsinized, centrifuged, and fixed with 2.5% glutaraldehyde in 0.2 M phosphate buffer (pH 7.2) for 12 h at 4°C, and post-fixed with 1% OsO4 in 0.2 M phosphate buffer (pH 7.2) for 2 h at 4°C. After washing with the phosphate buffer, the cells were dehydrated in a series of ethanol solutions of 30%, 50%, 70%, 95%, and 100% for 50 min, and then embedded with Epon 812 (Shell Chemical, London, UK) for polymerization. The embedded cells were sectioned to achieve a thickness of 75 nm using a Reichert ultramicrotome, and the samples were mounted onto 200 mesh copper grids. After negative staining with uranyl acetate and lead citrate for 5 min, the grids were visualized using an H600 transmission electron microscope (Hitachi Ltd, Chiyoda-ku, Japan) with an operating voltage of 60 kV.

### CT imaging of an atherosclerotic mice model

We used an apolipoprotein-E-gene-deficient (ApoE^-/-^) mice model of atherosclerosis in the experiment [35]. This animal model is generally accepted in the field. This model can imitate pathological changes in atherosclerotic lesions with high consistency when fed with a Western diet [35]. Animal experiments and animal care were carried out according to protocols approved by the Institutional Committee for Animal Care and in accordance with the policy of the National Ministry of Health. For CT imaging, ApoE^-/-^ mice (30 to 45 g, 1 year old, *n* = 8) purchased from the Shanghai Research Center for Model Organisms (Shanghai, China) were anesthetized by intraperitoneal injection of pentobarbital sodium (40 mg/kg). PEGylated Au DENPs (2.0 μmol Au/g body weight) dispersed in PBS buffer were then intravenously injected into the mice through the tail vein. The mice were scanned by a micro-CT imaging system (eXplore Locus, GE Healthcare, London, Ontario, Canada) with a tube voltage of 80 kV, an electrical current of 450 μA, and a slice thickness of 45 μm. CT scanning was performed both before and after intravenous injection of PEGylated Au DENPs (100 μL, [Au] = 0.1 M) at time points of 20 min, 2, 4, and 6 h post-injection, respectively. Images were reconstructed on a micro-CT imaging workstation using the following parameters: voxel, 45 × 45 × 45 μm; display field of view, 10 to 25 mm. CT values were acquired on the same workstation using the software supplied by the manufacturer.

### Histological characteristics and macrophage uptake of PEGylated Au DENPs

After micro-CT scanning, the animals were anesthetized and perfusion-fixation was performed. The heart, lung, liver, spleen, kidney, intestine, and vessel were harvested and immediately fixed in a 4% formalin solution. Then the organs were dehydrated and embedded with paraffin. The paraffin-embedded samples were cut into 5-μm sections using a conventional microtome. For silver staining, the sections were dewaxed, washed thoroughly in PBS, and stained with the silver enhancement kit according to the manufacturer's instructions. Sections were then washed with PBS and counterstained with 1% nuclear fast red. After washing thrice with PBS, the sections were then air-dried, dehydrated, and mounted onto cover slips for light microscope observation.

For HE staining, the pre-treated sample sections were dipped in a Coplin jar containing hematoxylin for 30 s. The samples were then rinsed with water for 1 min and stained with 1% eosin Y solution for 30 to 60 s. Finally, the cover slips were dehydrated and mounted onto glass slides before microscopic imaging.

### *In vivo* biodistribution of PEGylated Au DENPs

After CT imaging of macrophages in ApoE^-/-^ mice model of atherosclerosis at 6 h post-injection of PEGylated Au DENPs via intravenous injection, the mice were euthanized. Then, the heart, lung, stomach, spleen, liver, intestine, kidney, testicle, blood, and brain were extracted and weighed. The organs were cut into 1- to 2-mm^2^ pieces and incubated in aqua regia solution for 4 h. Au content was determined by a Leeman Prodigy inductively coupled plasma-atomic emission spectroscopy (ICP-AES) system (Teledyne Leeman Labs, Hudson, NH, USA).

### Statistical analysis

The data were expressed as mean values ± standard deviation (SD). For quantitative comparison and analysis, the values were evaluated by Student's *t*-test and a one-way analysis of variance (ANOVA) method. A probability value 0.05 was considered to indicate statistical significance, and the data were labeled with (*) for *p < 0.05* and (**) for *p < 0.01*.

## Results and discussion

### Synthesis of PEGylated Au DENPs

Our previous work [29] has shown that G5.NH_2_ dendrimers modified with *m*PEG-MAL (G5.NH_2_-*m*PEG) can be used as templates to entrap AuNPs with higher Au loading content and good stability. The modification of *m*PEG-MAL effectively improved the cytocompatibility of the formed PEGylated Au DENPs.

**Figure 1:**
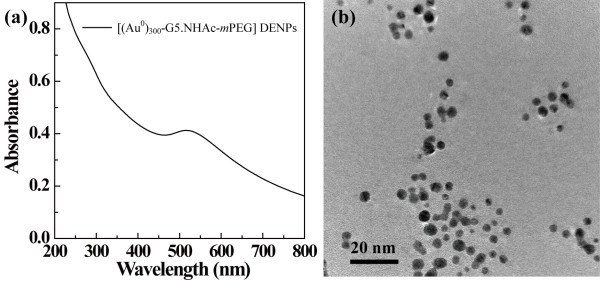
UV-vis spectrum (a) and TEM image (b) of PEGylated Au DENPs.

The PEGylated Au DENPs were prepared according to the experimental protocol reported in our previous report [29]. The formed PEGylated Au DENPs were characterized using UV-vis spectrometry and TEM (Figure 1). In Figure 1a, the PEGylated Au DENPs had a typical surface plasmon resonance (SPR) peak at around 515 nm, indicating the presence of AuNPs. TEM image demonstrated that the formed Au DENPs were relative uniform in morphology with a mean diameter of 3.2 nm (Figure 1b), in agreement with our previous report [29].

### Characterization of macrophages

**Figure 2:**
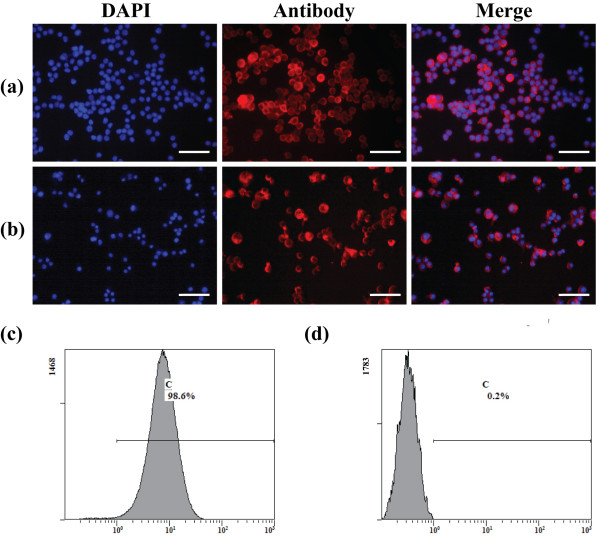
**Characterization of macrophages.** Immunofluorescence staining showed that more than 90% of the Ana-1 cells expressed the macrophage markers F4/80 (red, **a**) and MAC-3 (red, **b**) and the nuclei were stained with DAPI. Flow cytometry analysis of Ana-1 cells showed that F4/80 surface antigen was positive (98.6 ± 0.4) **(c)**, whereas the isotype control was negative **(d)**. Scale bar measures 100 μm.

Ana-1 cells adopted a more uniform round-shaped population (Figure 2a,b). Immunofluorescence staining showed that more than 99% of the Ana-1 cells expressed the macrophage special markers of F4/80 (Figure 2a) and MAC-3 (Figure 2b) and the cell nuclei were stained with DAPI. Phenotypic analysis by flow cytometry showed that the Ana-1 cells used in these experiments were strongly positive for macrophage surface antigen F4/80 (98.6 ± 0.2) (Figure 2c), whereas the isotype control was negative (Figure 2d).

### Cytotoxicity of PEGylated Au DENPs

**Figure 3:**
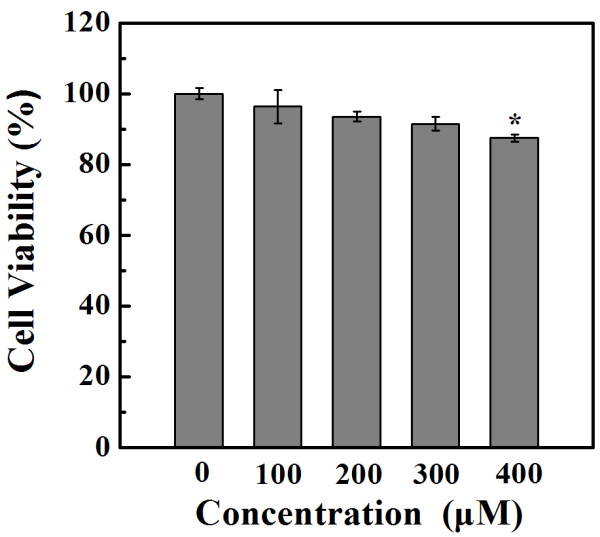
**CCK-8 assay of the viability of macrophages treated with PEGylated Au DENPs.** CCK-8 assay of the viability of macrophages treated with PEGylated Au DENPs with different concentrations for 24 h (*n* = 4).

Prior to using the PEGylated Au DENPs for molecular CT imaging, the *in vitro* cytotoxicity of the NPs to macrophages should be evaluated. The cytotoxicity of PEGylated Au DENPs was tested by CCK-8 assay of the viability of Ana-1 cells treated with the particles (Figure 3). Macrophages incubated with PEGylated Au DENPs at the Au concentration range of 100 to 300 μM had a similar cell viability compared with the untreated control cells (*p* > 0.05, *n* = 4). Only at the Au concentration up to 400 μM, the PEGylated Au DENPs started to display cytotoxicity. The non-cytotoxicity of PEGylated Au DENPs at Au concentration up to 300 μM suggests that PEGylation modification is a powerful strategy to significantly enhance the biocompatibility of Au DENPs for macrophage imaging applications.

Cell apoptosis is regarded as one of the most important characteristics for cytotoxicity [36]. To test the influence of PEGylated Au DENPs on macrophage apoptosis, the cells incubated with the particles were analyzed by flow cytometry (Additional file 1: Figure S1). The apoptotic index of Ana-1 cells treated with PEGylated Au DENPs at Au concentrations of 100 and 300 μM were estimated to be 3.3% ± 0.29% (Additional file 1: Figure S1b) and 3.57% ± 0.16% (Additional file 1: Figure S1c), respectively, with no statistically significant difference from that of the control cells treated with PBS (2.6% ± 0.27%, *p > 0.05*). This result suggests that PEGylated Au DENPs have no apoptotic effect on macrophage. Thus, the combined results of CCK-8 assay for cell cytotoxicity and flow cytometry analysis for cell apoptosis indicate that the PEGylated Au DENPs are non-cytotoxic in the given concentration range, which is essential for their uses in CT imaging of macrophages in atherosclerosis.

### *In vitro* micro-CT imaging

**Figure 4:**
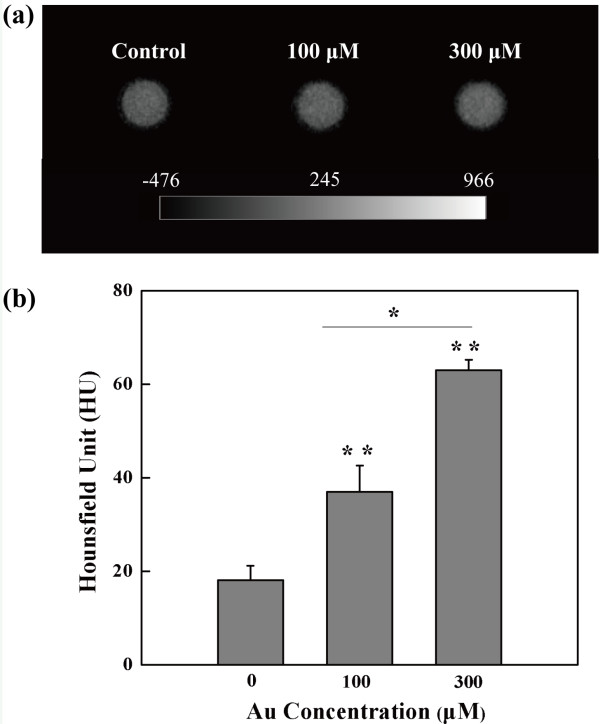
**Transverse micro-CT images and CT values of the Ana-1 cell suspensions.** Transverse micro-CT images **(a)** and CT values **(b)** of the Ana-1 cell suspensions incubated with PEGylated Au DENPs with different Au concentrations for 24 h (*n* = 3).

To prove that PEGylated Au DENPs enable CT imaging of macrophages *in vitro*, Ana-1 cells incubated with PEGylated Au DENPs with two different concentrations (100 and 300 μM) for 4 h were imaged (Figure 4a). Given the fact that it is difficult to visually distinguish the brightness of CT images of macrophages treated with Au DENPs, quantitative analysis of the CT signal intensity is necessary. Quantitative analysis of the CT values of macrophages (Figure 4b) showed that the CT value of Ana-1 cells incubated with PEGylated Au DENPs at the Au concentration of 300 μM was significantly higher than that of the cells incubated at a lower concentration (100 μM) (*p* < 0.05) and that of the negative control cells (*p* < 0.01). The higher CT values of macrophages at a higher concentration of PEGylated Au DENPs (300 μM) may be associated with the cellular uptake of more particles, which enable CT imaging of macrophages *in vitro*.

### Cellular uptake of PEGylated Au DENPs

**Figure 5:**
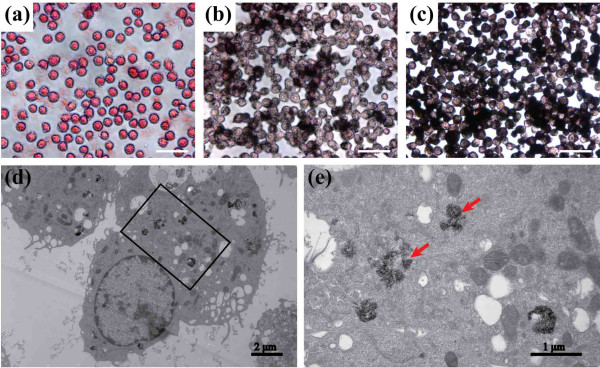
**Representative images of Ana-1 cells subjected to silver staining and TEM. (a)** Negative control cells treated with PBS buffer and the cells incubated with PEGylated Au DENPs (at the Au concentration of 100 μM **(b)** and 300 μM **(c)** for 24 h). The image **(d)** shows TEM images of cells incubated with PEGylated Au DENPs at the Au concentration of 300 μM for 24 h; and the image **(e)** shows the magnified view of the square area in **(d)**. The black arrow in **(e)** shows the domain of Au DENPs. Scale bar measures 100 μm.

The uptake of PEGylated Au DENPs in the Ana-1 cells was confirmed by silver staining. As shown in Figure 5, accumulated black spots of silver staining were found in the cytoplasm of Ana-1 cells treated with PEGylated Au DENPs (at the Au concentration of 100 μM (b) and 300 μM (c)) for 24 h. The color of the spots became darker at a higher Au concentration, which implies the increase in cellular uptake of PEGylated Au DENPs. In contrast, the Ana-1 cells treated with PBS did not show any black spots (Figure 5a).

TEM imaging of macrophages treated with PEGylated Au DENPs was also performed to identify the particle distribution in subcellular structures. After incubation with PEGylated Au DENPs (300 μM) for 24 h, numerous high electron-staining particles were found in the cytoplasm of cells (Figure 5d,e), especially in the lysosomes or phagocytic vacuoles. By sharp contrast, no electron-staining particles were observed in the cytoplasm of the control Ana-1 cells treated with PBS (Additional file 2: Figure S2). The TEM results show that PEGylated Au DENPs were phagocytized by macrophages instead of adhering to the surface of cells. The internalization of PEGylated Au DENPs likely occurs through two different mechanisms, such as phagocytosis and diffusion via cell walls, in agreement with previous literature reports [37,38]. The TEM data also verify that the incubation of PEGylated Au DENPs with a high Au concentration (300 μM) do not significantly affect the morphology and subcellular structures of cells, corroborating the CCK-8 and cell apoptosis assay data.

### CT imaging of an atherosclerotic mice model

**Figure 6:**
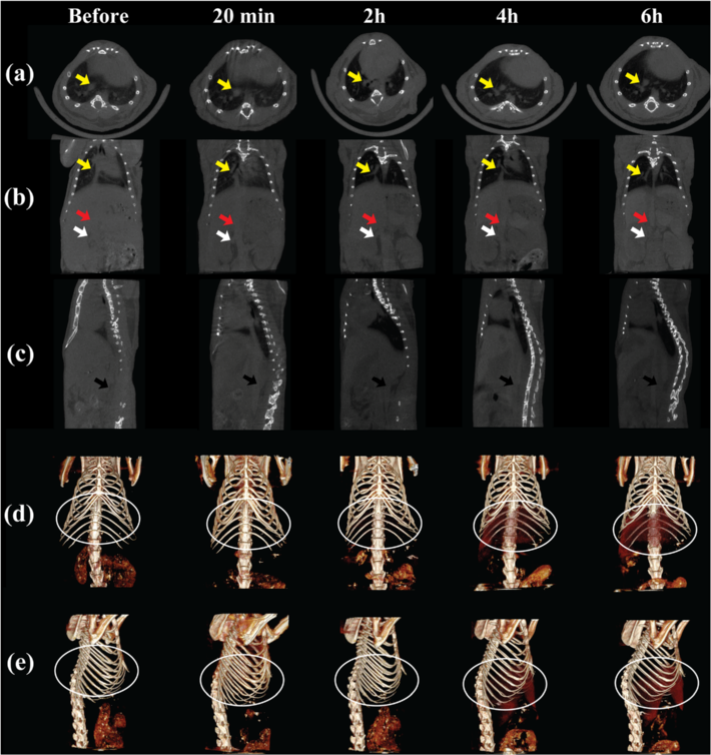
**Representative *in vivo *micro-CT images of macrophages in ApoE**^**-/- **^**mice model of atherosclerosis.** Representative *in vivo* micro-CT images of macrophages in ApoE^-/-^ mice model of atherosclerosis before and after intravenous injection with PEGylated Au DENPs (100 μL, [Au] = 0.1 M) for 20 min, 2, 4, and 6 h. **(a)** Transverse planes, **(b)** coronal planes, **(c)** sagittal planes, and corresponding 3D renderings of *in vivo* CT images **(d, e)**.

We next used the PEGylated Au DENPs for CT imaging of an atherosclerotic mice model. ApoE^-/-^ mice were intravenously injected with PBS solutions containing PEGylated Au DENPs ([Au] = 0.1 M, 100 μL). The micro-CT images of a mouse at a transverse section, coronal plane, sagittal plane, and 3D reconstructed images are shown in Figure 6. The pulmonary artery (PA) pointed by a yellow arrow (Figure 6a,b), IVC of the mice pointed by a red arrow (Figure 6b,c), and abdominal aorta (AA) of the mice pointed by a black arrow (Figure 6b,c) can be clearly seen. In a coronal plane of the micro-CT images (Figure 6b), the white arrow points to the renal vein, while the red arrow points to the IVC. In the sagittal plane of the micro-CT images (Figure 6c), the black arrow points to the AA, and it is clear that the vessels show an apparent enhancement after administration of PEGylated Au DENPs with a CT value higher than that before injection. Our results show that with the post-injection time, PEGylated Au DENPs may be able to diffuse into the entire macrophage area and are phagocytized by the macrophages. Thus, the vessels gradually became clearer and more defined. In addition, the liver regions of the mice were also be easily discerned at 4 and 6 h post-administration of PEGylated Au DENPs in the reconstructed 3D CT images (Figure 6d,e). Our results suggest that modification of Au DENPs with PEG molecules can prolong their blood circulation time in the ApoE^-/-^ mice by decreasing the rapid uptake and clearance in the RES.

**Figure 7:**
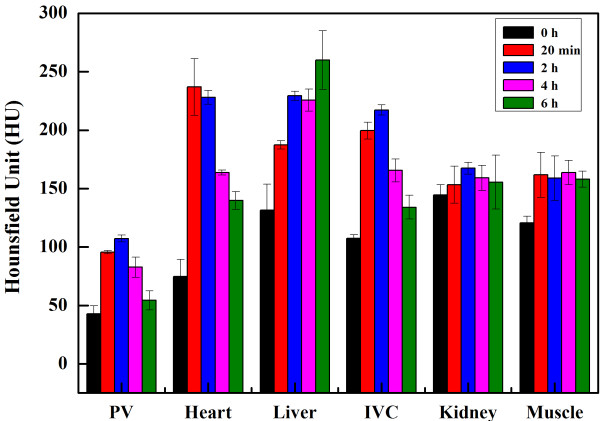
**CT values (HU) of different organs before injection and at different time points.** CT values (HU) of different organs before injection and at different time points post-intravenous injection of PEGylated Au DENPs (100 μL, [Au] = 0.1 M). Abbreviation: pulmonary vein (PA), inferior vena cava (IVC).

Careful analysis of the CT values of the macrophage area at different time points shows that even at 6 h post-injection of the PEGylated Au DENPs, the CT values of the macrophage areas are still significantly higher than those of the macrophage areas before injection. In contrast to the CT values of PA, heart, liver, and IVC, which all display a significant increase after injection of PEGylated Au DENPs, the CT values in the muscle and kidney areas are slightly similar to those before the injection of the particles (Figure 7). Therefore, part of the PEGylated Au DENPs could be transported to the macrophage sites and be ingested by macrophages [39,40], enabling effective CT imaging of blood pool in the atherosclerotic mice, which is highly important for early stage diagnosis of atherosclerotic diseases.

### *In vivo* macrophage uptake of PEGylated Au DENPs

**Figure 8:**
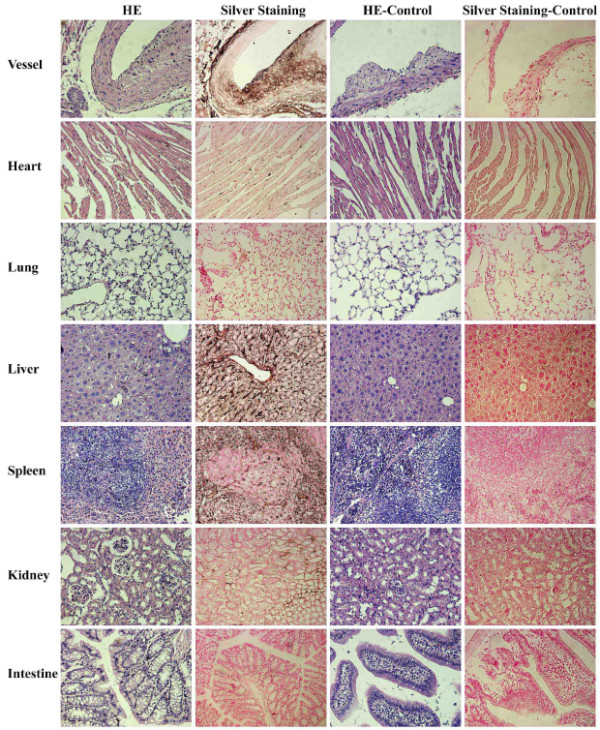
**Representative HE staining and silver staining of main visceral organs in ApoE**^**-/- **^**mice.** Representative HE staining and silver staining of main visceral organs in ApoE^-/-^ mice before (control) and after intravenous injection with PEGylated Au DENPs for 6 h.

To investigate the safety of PEGylated Au DENPs ([Au] = 0.1 M, 100 μL) in ApoE^-/-^ mice and also the impact of the particles on the tissue structures, the major organs of the mice were extracted after 6 h post-injection of the PEGylated Au DENPs. Then HE staining and silver staining of the tissue sections from the extracted major organs were performed and observed using optical microscopy. HE staining (Figure 8) results reveal that the structure and morphology of the major organ tissues do not have apparent changes after intravenous injection of the PEGylated Au DENPs when compared with the control groups before injection. These results suggest that the PEGylated Au DENPs ([Au] = 0.1 M, 100 μL) are safe and do not affect the biological function of the organs. From silver staining results, it can be seen that after about 6 h of metabolism, Au DENPs (black spots) are able to be identified mainly in the vessel, liver, and spleen regions, with significantly smaller uptake in the lung, kidney, heart, and intestines.

### Biodistribution of PEGylated Au DENPs

**Figure 9:**
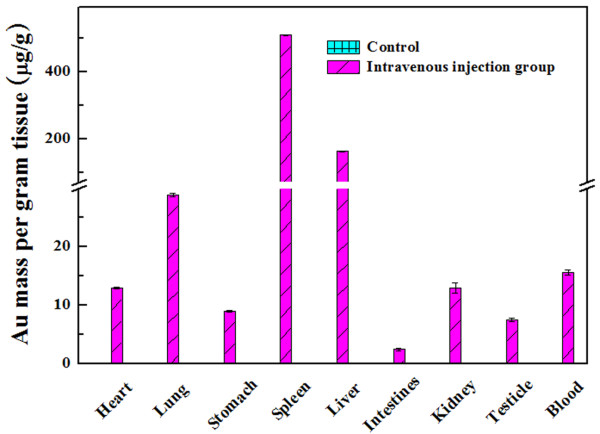
**Biodistribution of PEGylated Au DENPs in different organs.** Data were obtained by ICP-AES at 6 h post-injection in an ApoE^-/-^ mice model.

The macrophage uptake and biodistribution of PEGylated Au DENPs were analyzed after 6 h post-intravenous injection. ICP-AES was performed to quantify the Au concentration in the major organs such as heart, lung, stomach, spleen, liver, intestine, kidney, testicle, blood, and brain in the intravenous injection group with PEGylated Au DENPs and the control group without PEGylated Au DENP treatment (Figure 9). The results reveal that the spleen has the most significant Au uptake. After 6 h post-intravenous injection, the relatively high uptake of Au in the blood suggests that the PEGylated Au DENPs are able to escape from the RES located in the liver and pass through the renal filter, thereby enhancing their circulation time in the ApoE^-/-^ mice. Detailed pharmacokinetic investigations of the PEGylated Au DENPs at extended time points are necessary for a complete understanding of the metabolism and biodistribution of the Au DENPs.

## Conclusions

In summary, PEGylated Au DENPs were synthesized and used as contrast agents for CT imaging of macrophages associated with atherosclerosis *in vitro* and *in vivo*. The combined results of CCK-8 assay and flow cytometry analysis show that the PEGylated Au DENPs are non-cytotoxic at the Au concentration up to 300 μM. Silver staining and TEM data confirm that the PEGylated Au DENPs can be efficiently phagocytized and are mainly located in the lysosomes of macrophages. Further *in vitro* CT imaging of Ana-1 cells treated with the PEGylated Au DENPs and *in vivo* blood pool CT imaging of ApoE^-/-^ mice intravenously injected with the particles indicates that the developed PEGylated Au DENPs are able to image macrophages *in vitro* and the atherosclerotic mice *in vivo*. Findings from our study suggest that the developed Au DENPs have a great potential to be used as contrast agents for CT imaging of blood pool in atherosclerotic mice, which is important for the understanding of the pathological physiology of cardiovascular disease.

## Competing interests

The authors declare that they have no competing interests.

## Authors' contributions

KY and JQ carried out the culture and characterization of macrophages’ studies, participated in the sequence alignment, and drafted the manuscript. CP carried out the design and synthesis of Au DENPs, ZP carried out the immunoassays. XY participated in the sequence alignment. LH participated in the design of the study, and FY performed the statistical analysis. MJ and XL conceived of the study and participated in its design and coordination. All authors read and approved the final manuscript.

## Supplementary Material

Additional file 1: Figure S1 Flow cytometry analysis of apoptosis of macrophages treated without PEGylated Au DENPs **(a)** or with PEGylated Au DENPs at Au concentration of 100 μM **(b)** and 300 μM **(c)** for 24 h (*n* = 4). Click here for file

Additional file 2: Figure S2 Representative TEM images of Ana-1 cells without treatment with PEGylated Au DENPs** (a)**. The image **(b)** shows the magnified view of the square area in (a). Click here for file
