# Supplementary figures and images for: Polyethylene glycol-modified dendrimer-entrapped gold nanoparticles enhance CT imaging of blood pool in atherosclerotic mice
Source: Nanoscale Res Lett. 2014 Sep 26;9(1):529. doi: 10.1186/1556-276X-9-529 (PMC4184899; doi:10.1186/1556-276X-9-529)

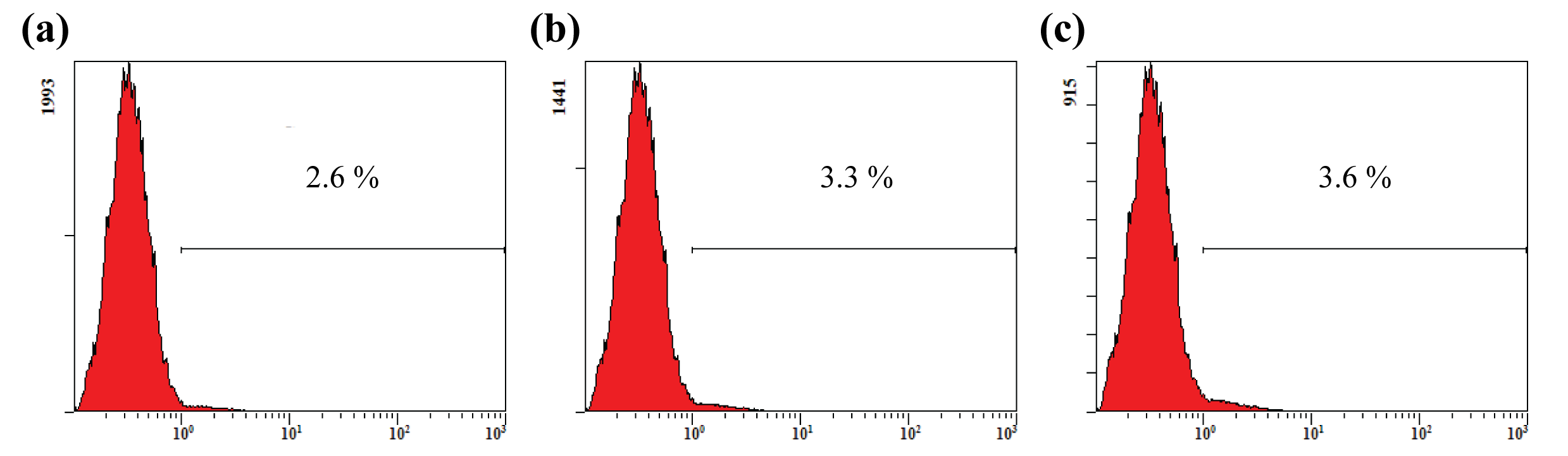

Supplement: Additional file 1: Figure S1 — Flow cytometry analysis of apoptosis of macrophages treated without PEGylated Au DENPs (a) or with PEGylated Au DENPs at Au concentration of 100 μM (b) and 300 μM (c) for 24 h (n = 4). [file 1556-276X-9-529-S1.tiff]

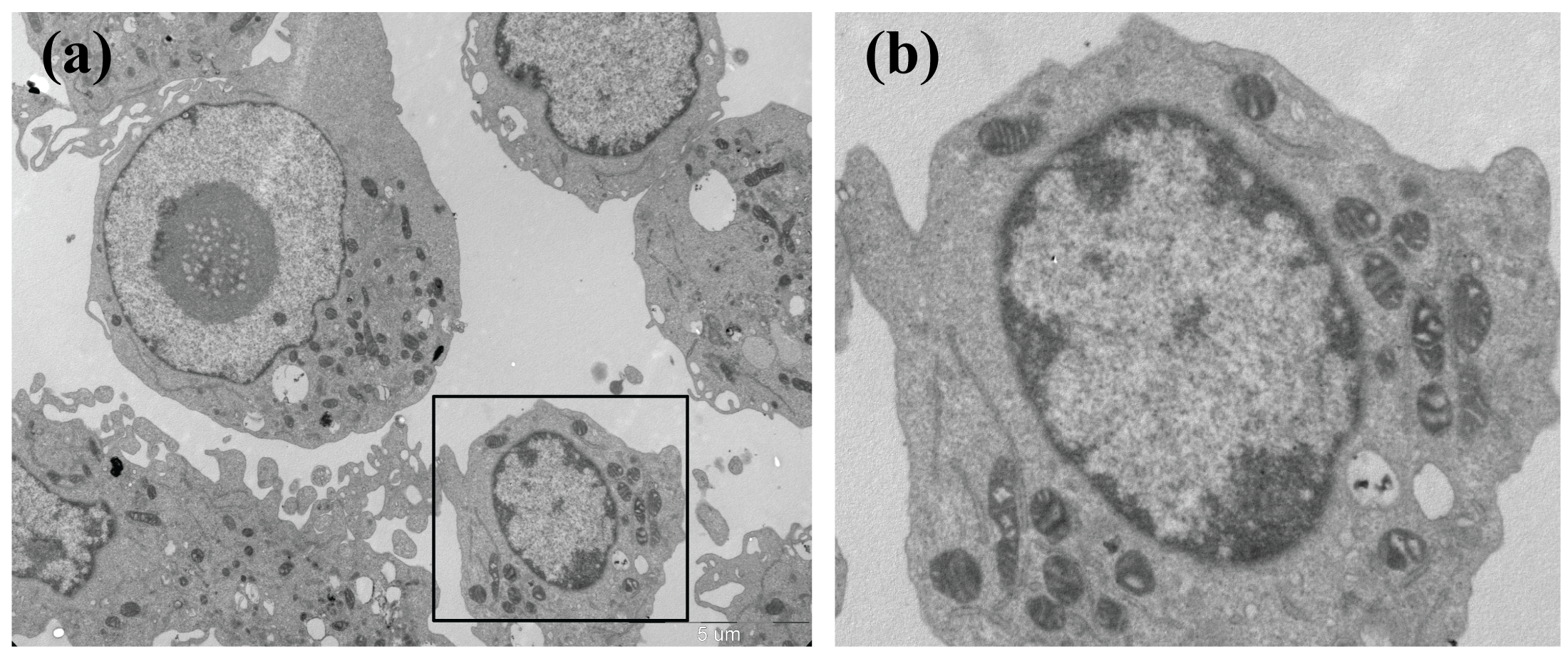

Supplement: Additional file 2: Figure S2 — Representative TEM images of Ana-1 cells without treatment with PEGylated Au DENPs (a). The image (b) shows the magnified view of the square area in (a). [file 1556-276X-9-529-S2.tiff]
